# Supplementary figures and images for: PP2A/B55α substrate recruitment as defined by the retinoblastoma-related protein p107
Source: eLife. 2021 Oct 18;10:e63181. doi: 10.7554/eLife.63181 (PMC8575462; doi:10.7554/eLife.63181)

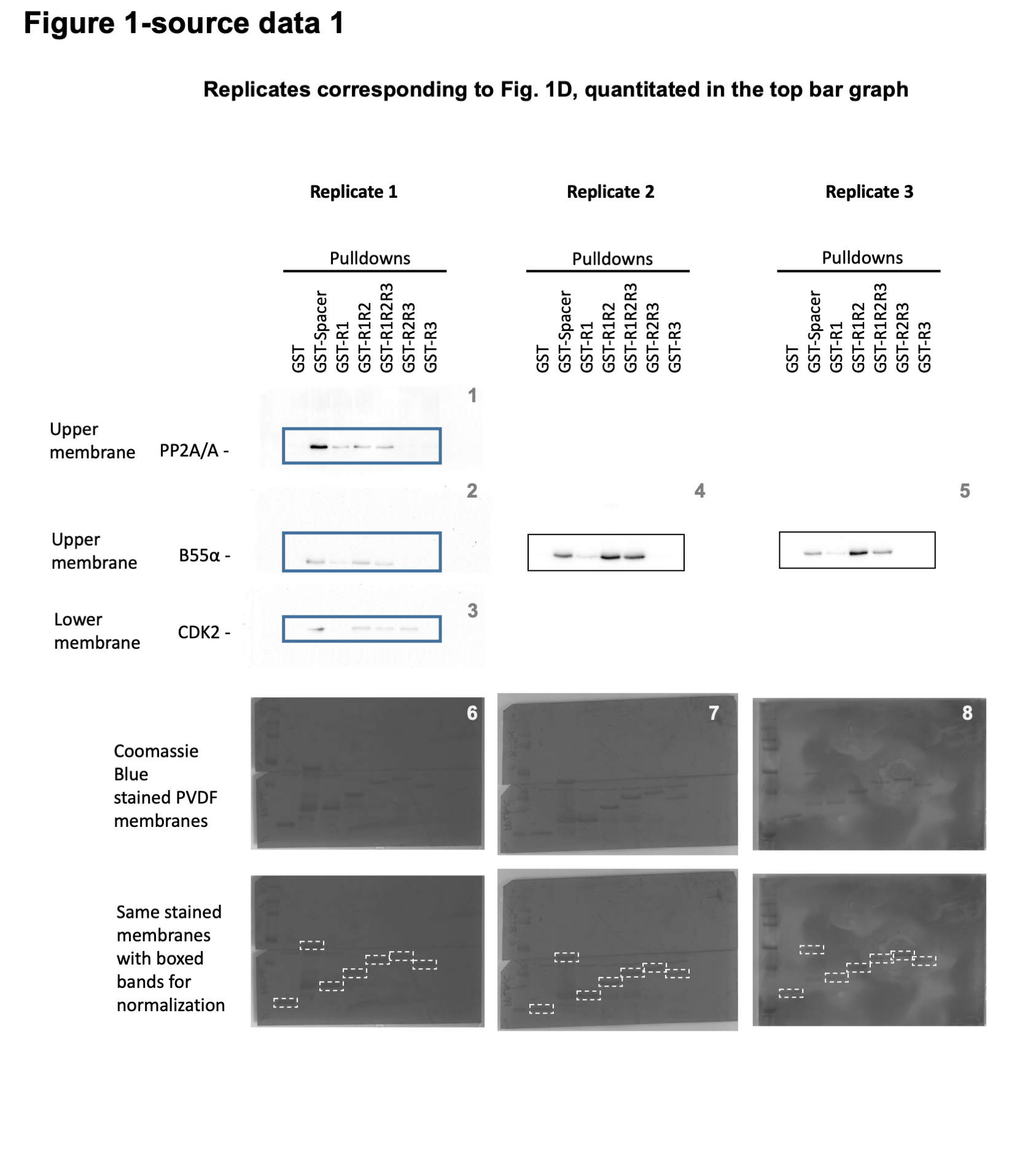

Supplement: Figure 1—source data 1. — B55α signal was normalized to the GST-fusion protein signal (selected bands are marked with dashed white boxes and corrected for background from a band-less identical area). [file elife-63181-fig1-data1.zip › 789c62da-bff6-4938-861c-77aebee5c21b.tiff]

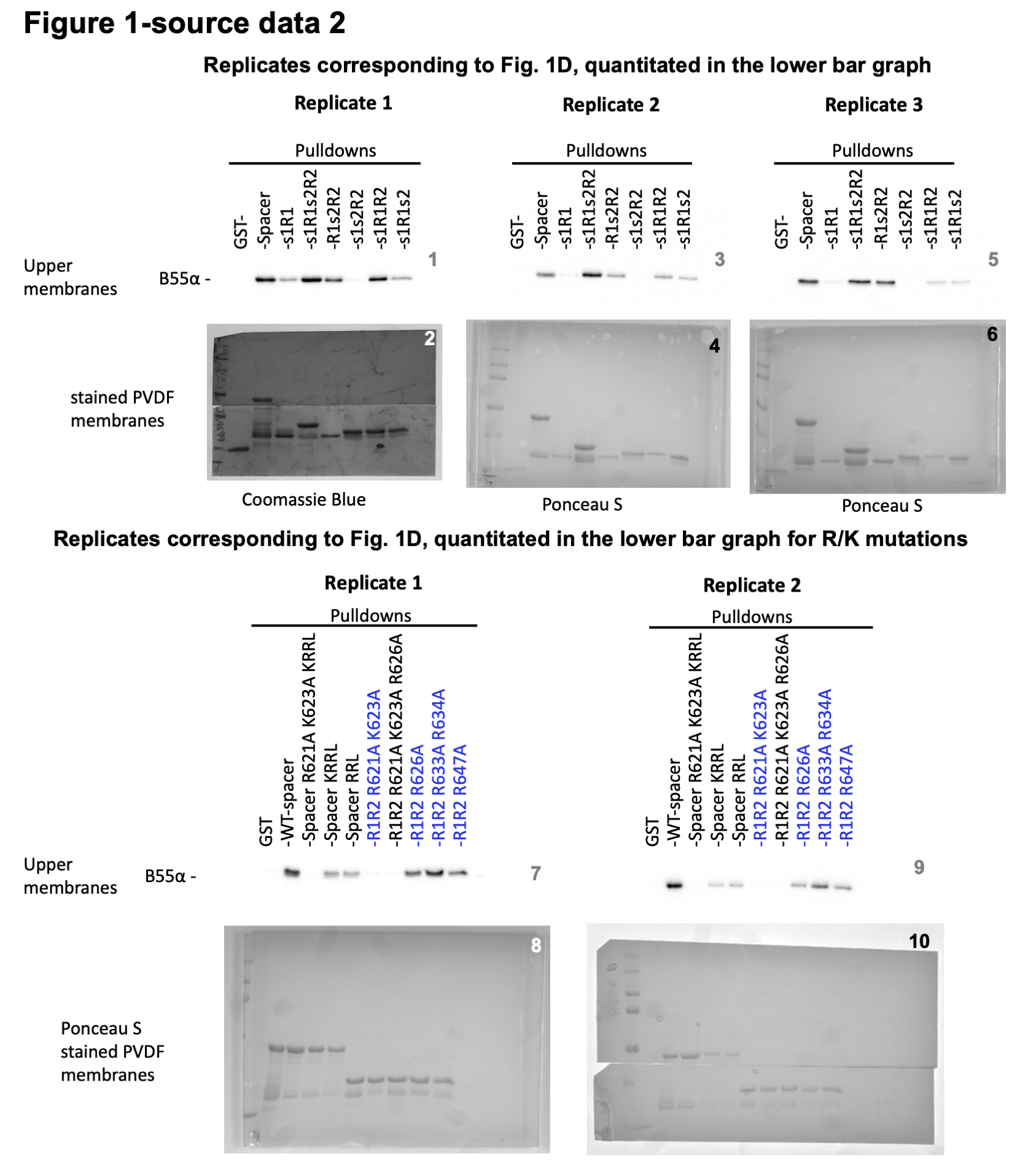

Supplement: Figure 1—source data 2. — Only the R/K mutants labeled blue were included in the quantitation. [file elife-63181-fig1-data2.zip › a323d102-3bc6-41d2-a179-89b83c5e7045.tiff]

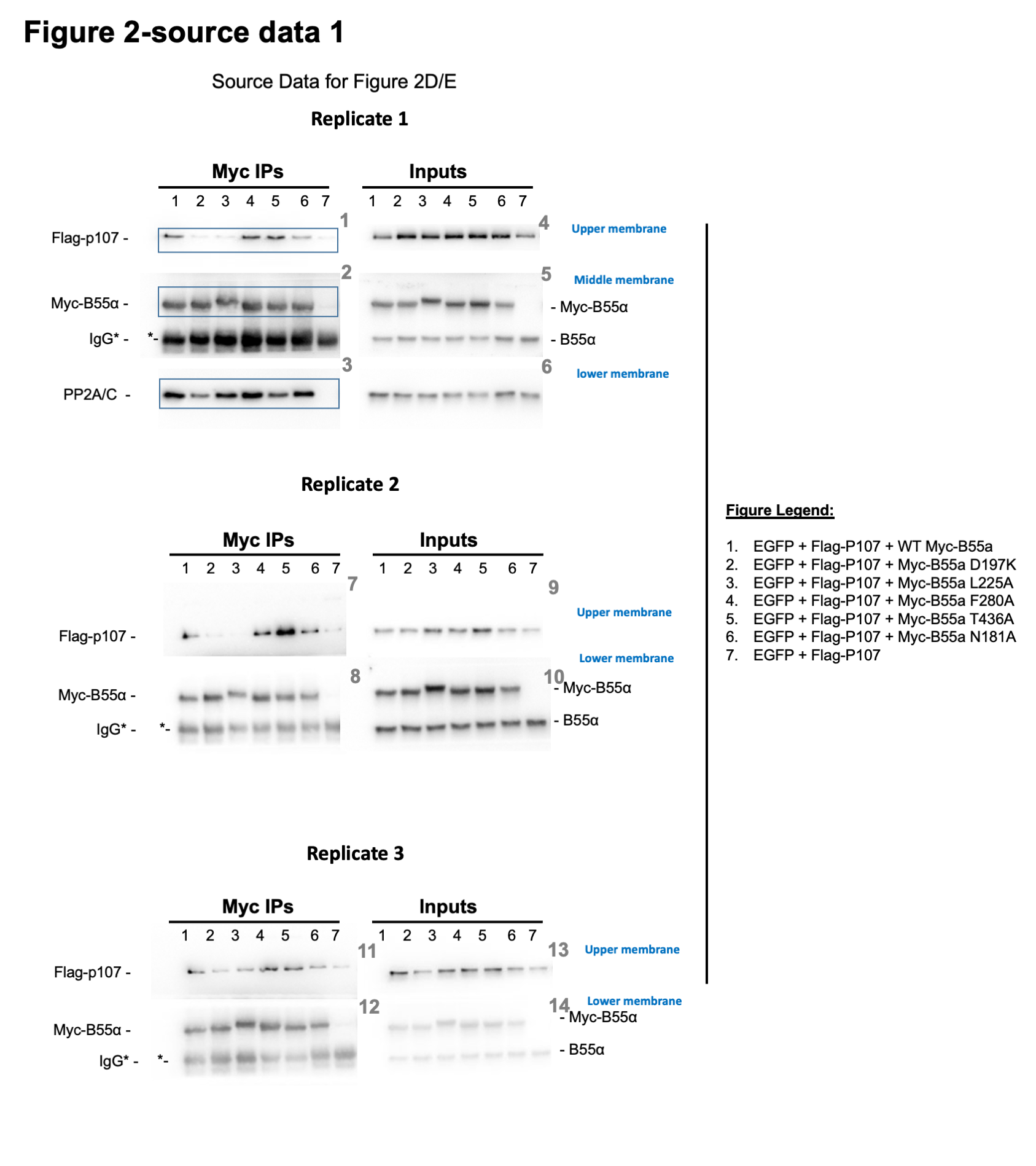

Supplement: Figure 2—source data 1. — Boxes indicate approximate area shown in Figure 2D. Western blot membranes for replicates 2 and 3. All replicates were used for the quantitation shown in Figure 2E. The legend indicates the B55α variants used in this set of replicates. Relevant proteins and IgG (in the IP membranes) are indicated. [file elife-63181-fig2-data1.zip › 581d8d01-0f78-4594-b086-f94276b8f597.tiff]

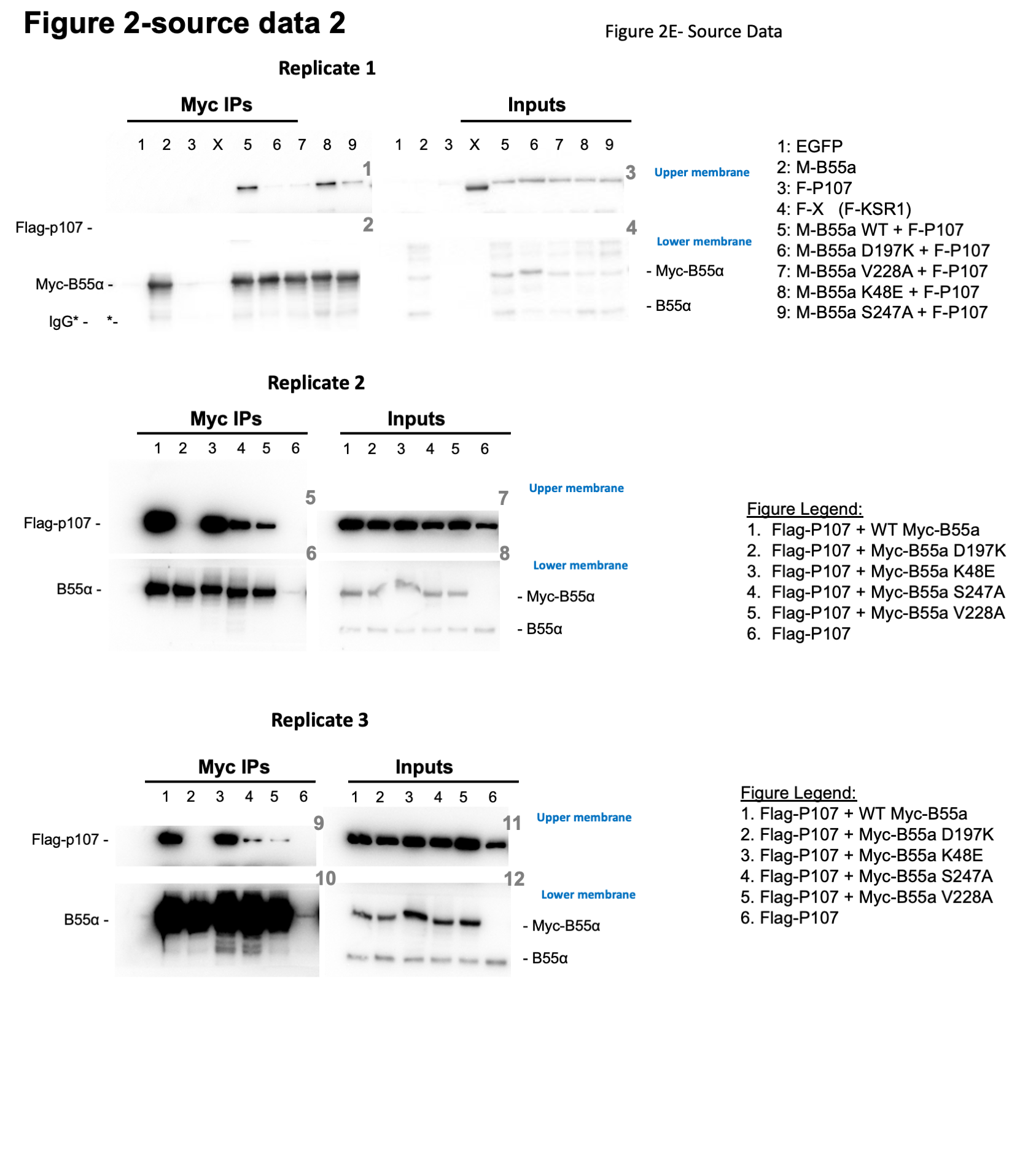

Supplement: Figure 2—source data 2. — All replicates were used for the quantitation shown in Figure 2E. The legends indicate the B55α variants used in this set of replicates. Relevant proteins and IgG (in the IP membranes) are indicated. [file elife-63181-fig2-data2.zip › a2d4776e-942b-459a-9f7c-32cec5bccb34 (1).tiff]

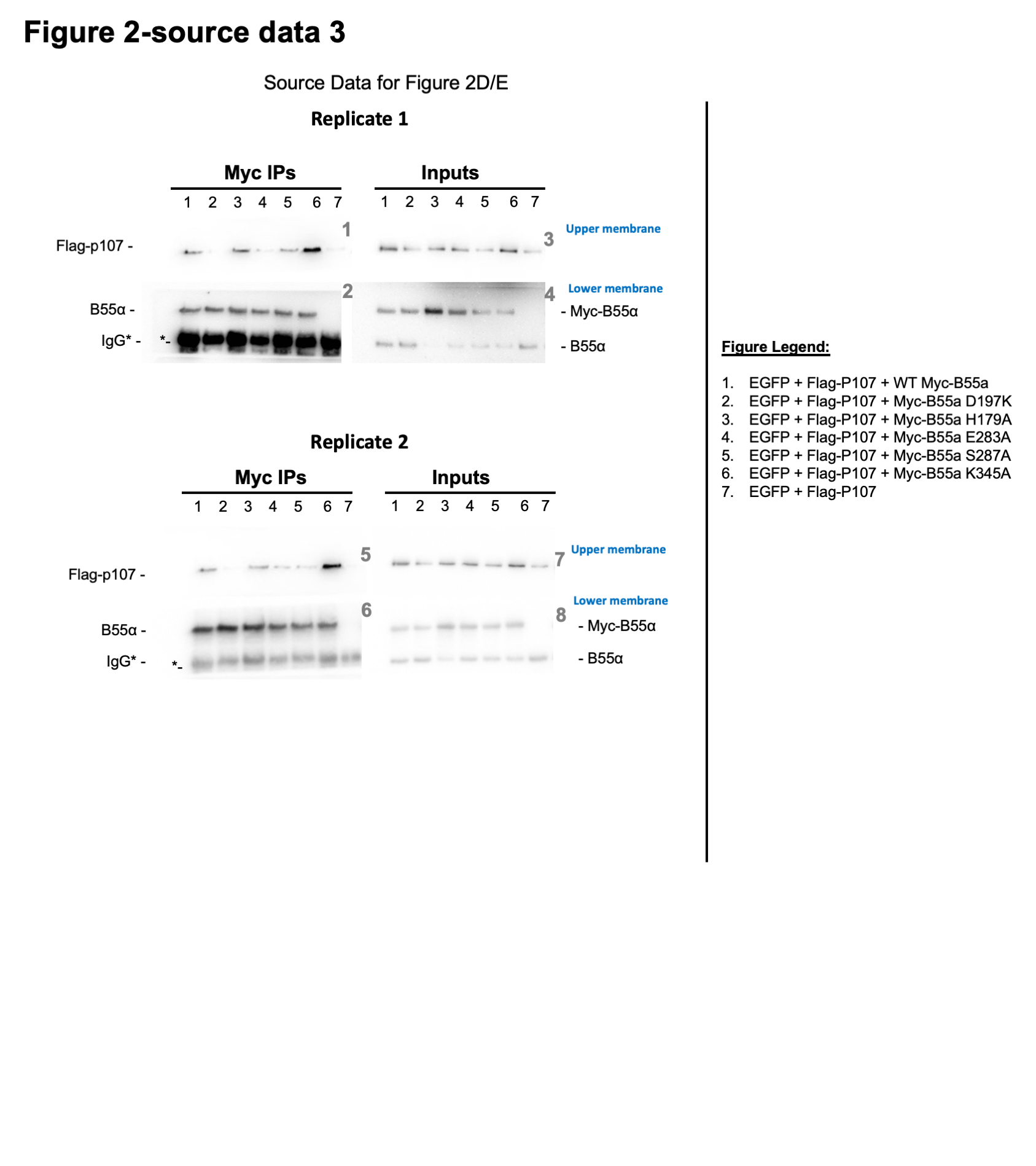

Supplement: Figure 2—source data 3. — The legend indicates the B55α variants used in this set of replicates. Relevant proteins and IgG (in the IP membranes) are indicated. [file elife-63181-fig2-data3.zip › 4b26a251-2c55-41a6-8bfe-a482a557a467.tiff]

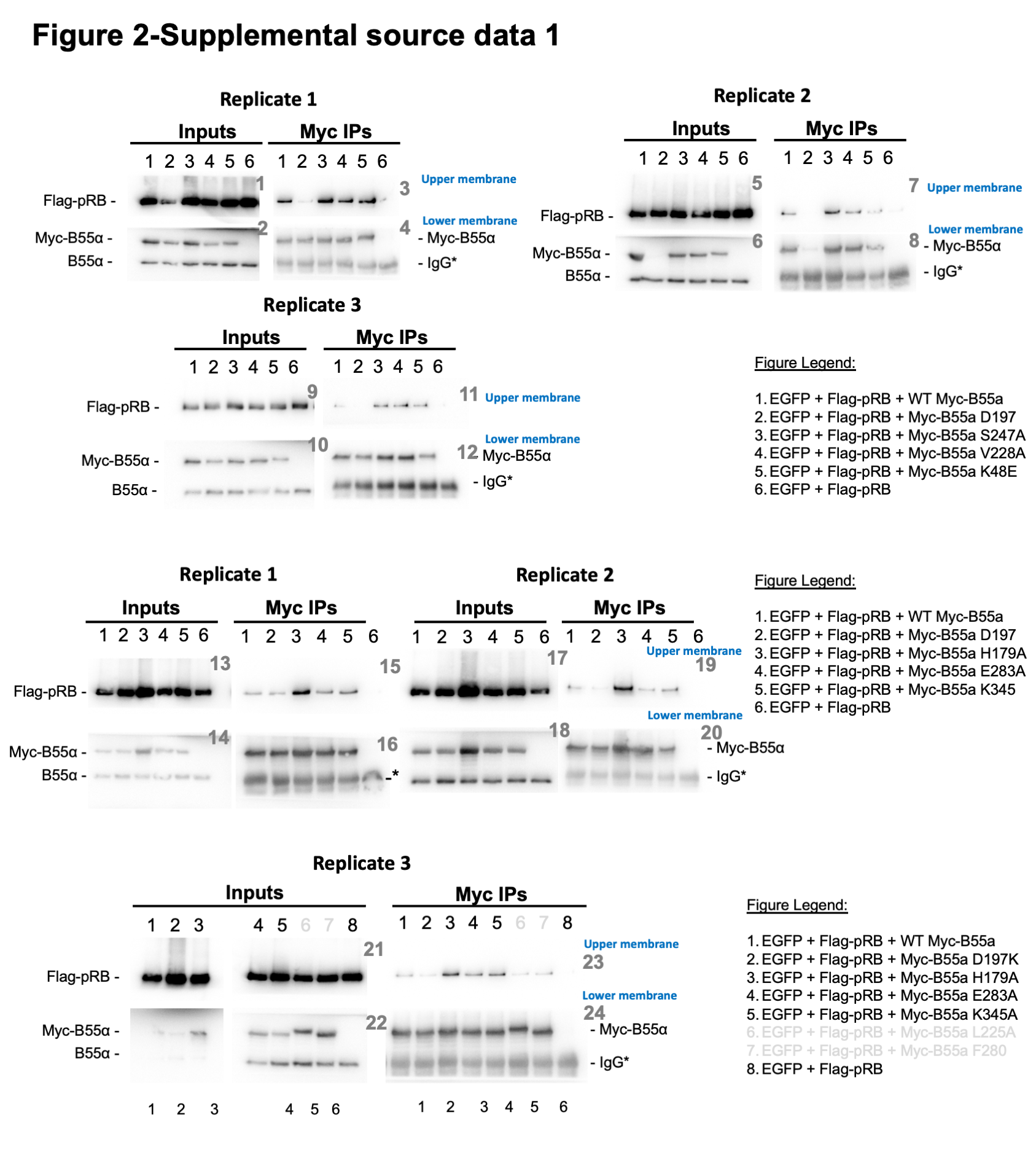

Supplement: Figure 2—figure supplement 1—source data 1. — The legends indicate the B55α variants used in this set of replicates. Relevant proteins and IgG (in the IP membranes) are indicated. [file elife-63181-fig2-figsupp1-data1.zip › 11539242-851b-4243-a609-82ee9a17f608.tiff]

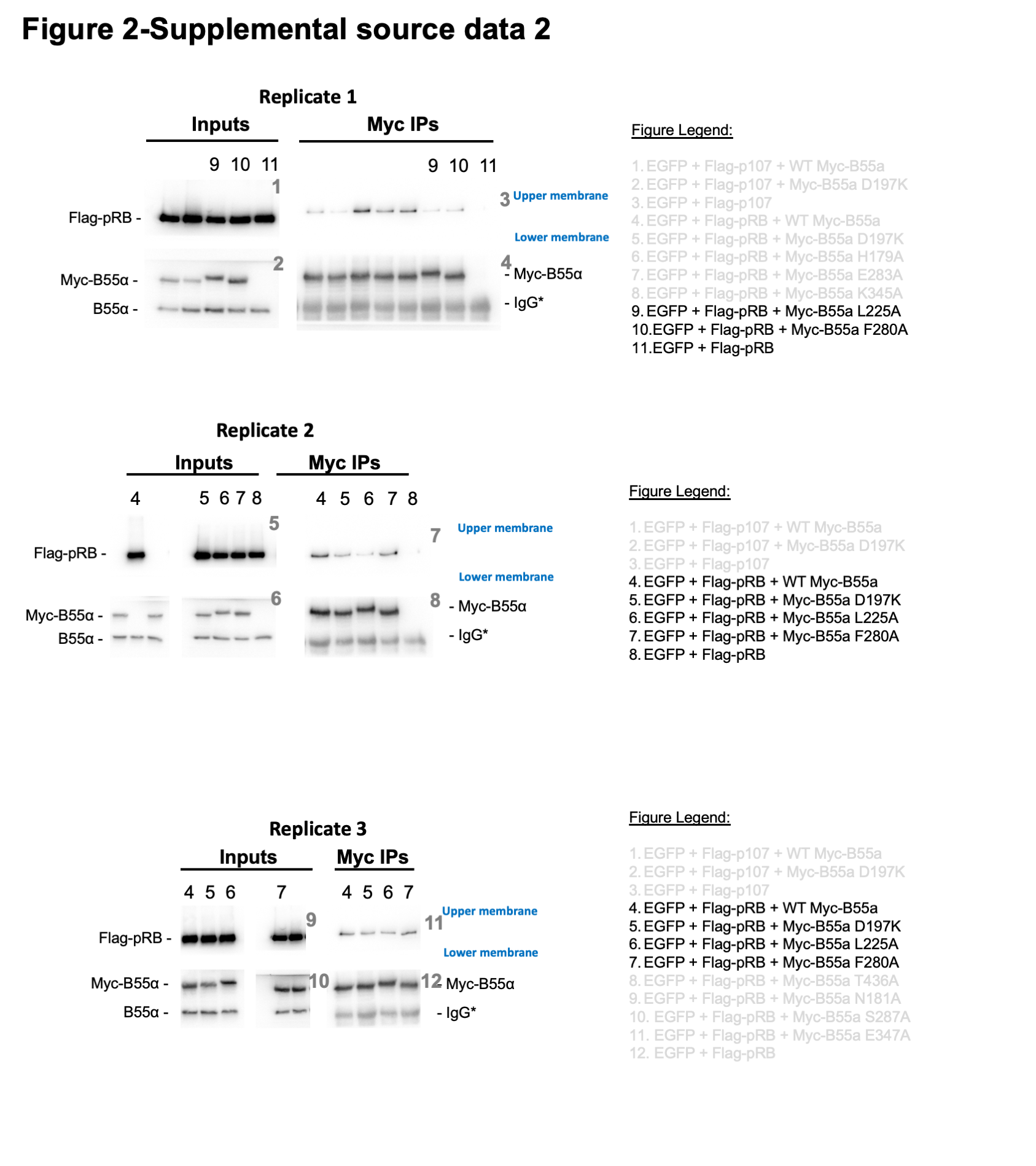

Supplement: Figure 2—figure supplement 1—source data 2. — The legends indicate the B55α variants used in this set of replicates. Relevant proteins and IgG (in the IP membranes) are indicated. [file elife-63181-fig2-figsupp1-data2.zip › 05820d41-4f3f-4ba3-86f7-8e11ce44abd8.tiff]

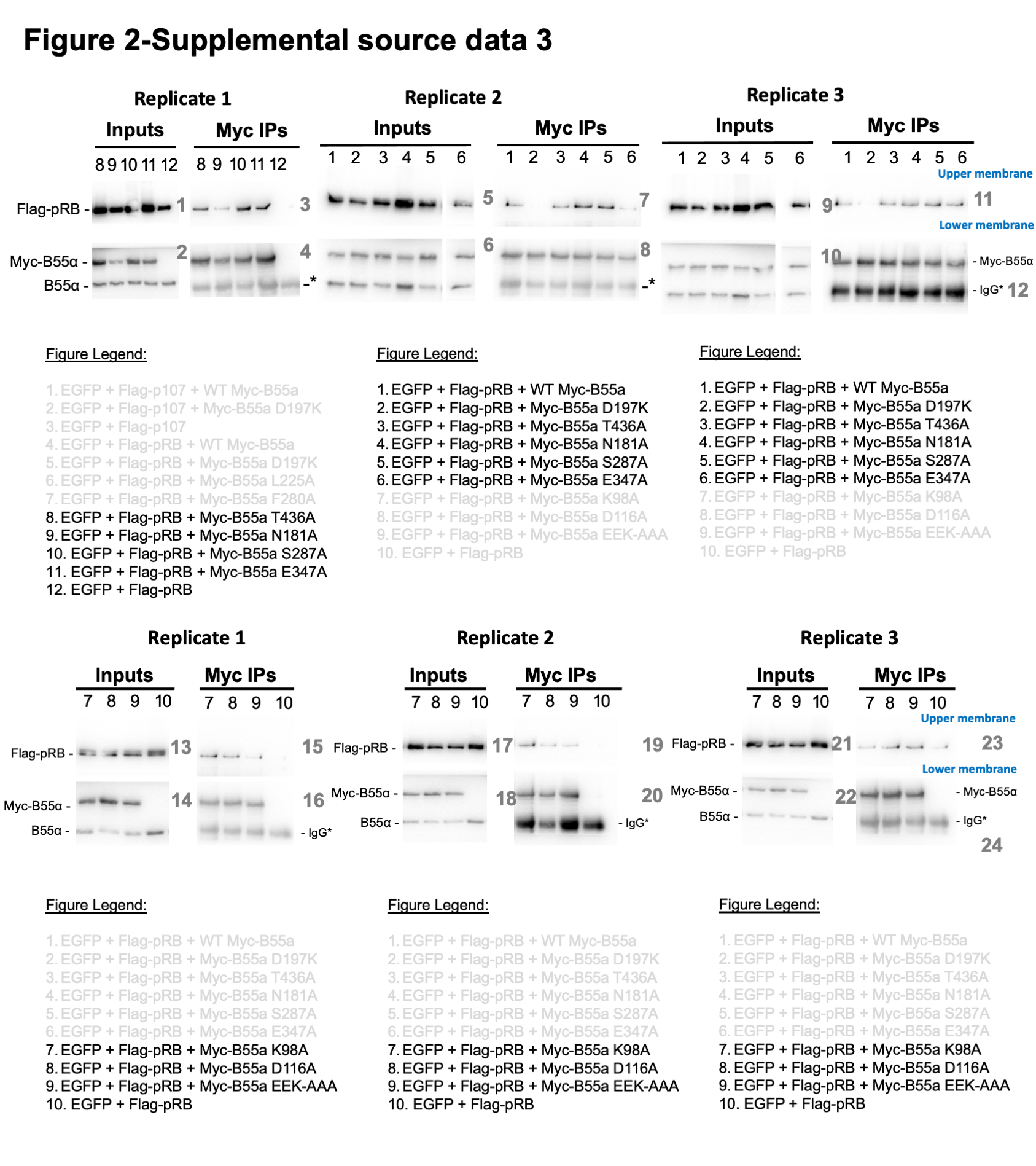

Supplement: Figure 2—figure supplement 1—source data 3. — The legends indicate the B55α variants used in this set of replicates. Relevant proteins and IgG (in the IP membranes) are indicated. [file elife-63181-fig2-figsupp1-data3.zip › c79c2670-38ec-4f50-b646-45dae5688fec.tiff]

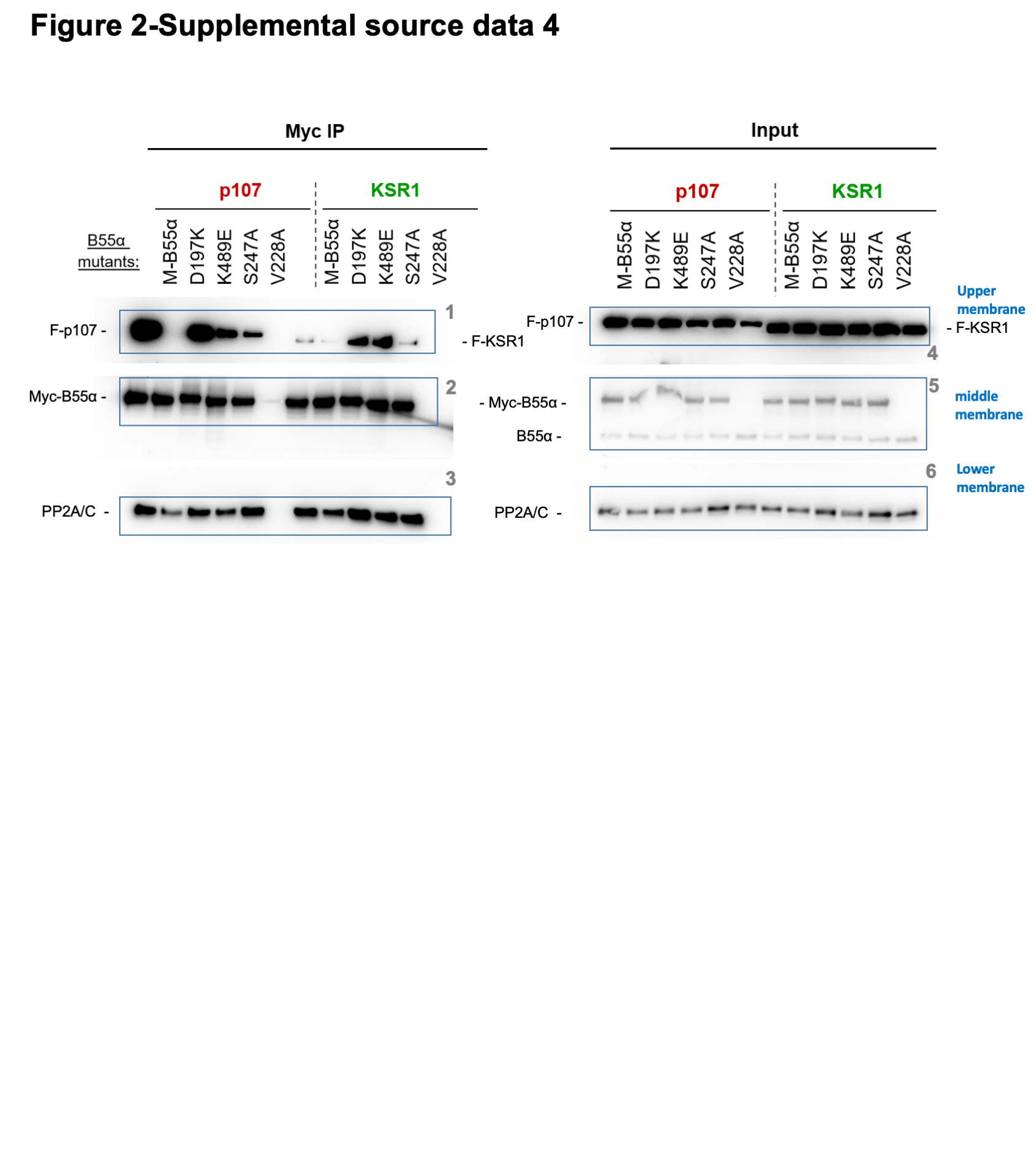

Supplement: Figure 2—figure supplement 1—source data 4. — Boxes indicate approximate area shown in the figure. Relevant proteins are indicated. [file elife-63181-fig2-figsupp1-data4.zip › 25a6d237-28c3-45fa-9ca7-505fd2b6be98.tiff]

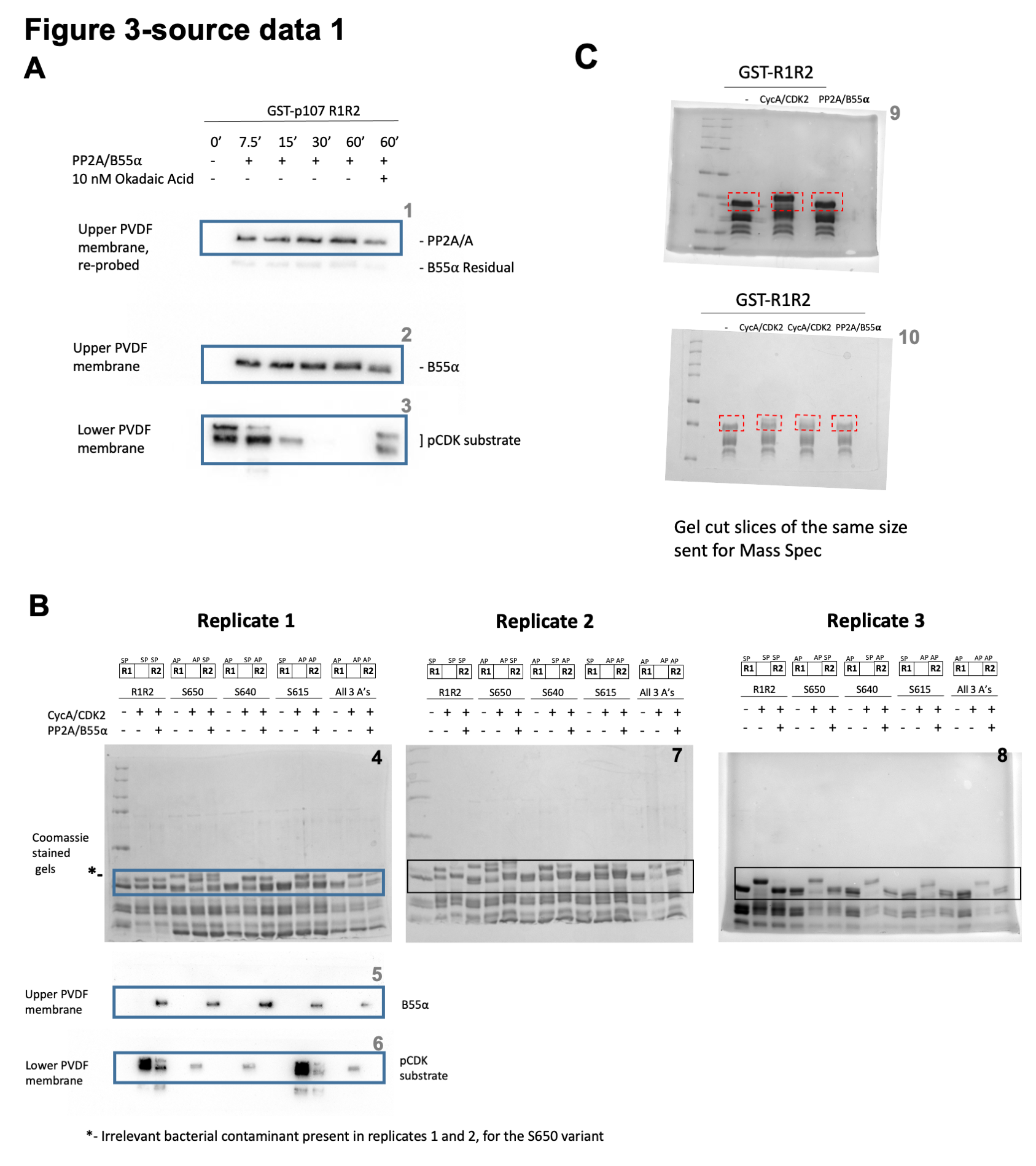

Supplement: Figure 3—source data 1. — Images in Figure 3A were generated from the boxed regions in each PVDF membrane. Comparable experiments are shown in Figure 4. Images in Figure 3B were generated from the boxed regions in replicate 1. Replicates 1–3 show comparable dephosphorylation of WT and MT p107 R1R2 by Coomassie Blue staining. The gel image in Figure 3C was generated form representative replicate 1, and the bands cut out for mass spectrometry are boxed. [file elife-63181-fig3-data1.zip › 652dbd2e-1044-43e3-93b5-925a9392f14d.tiff]

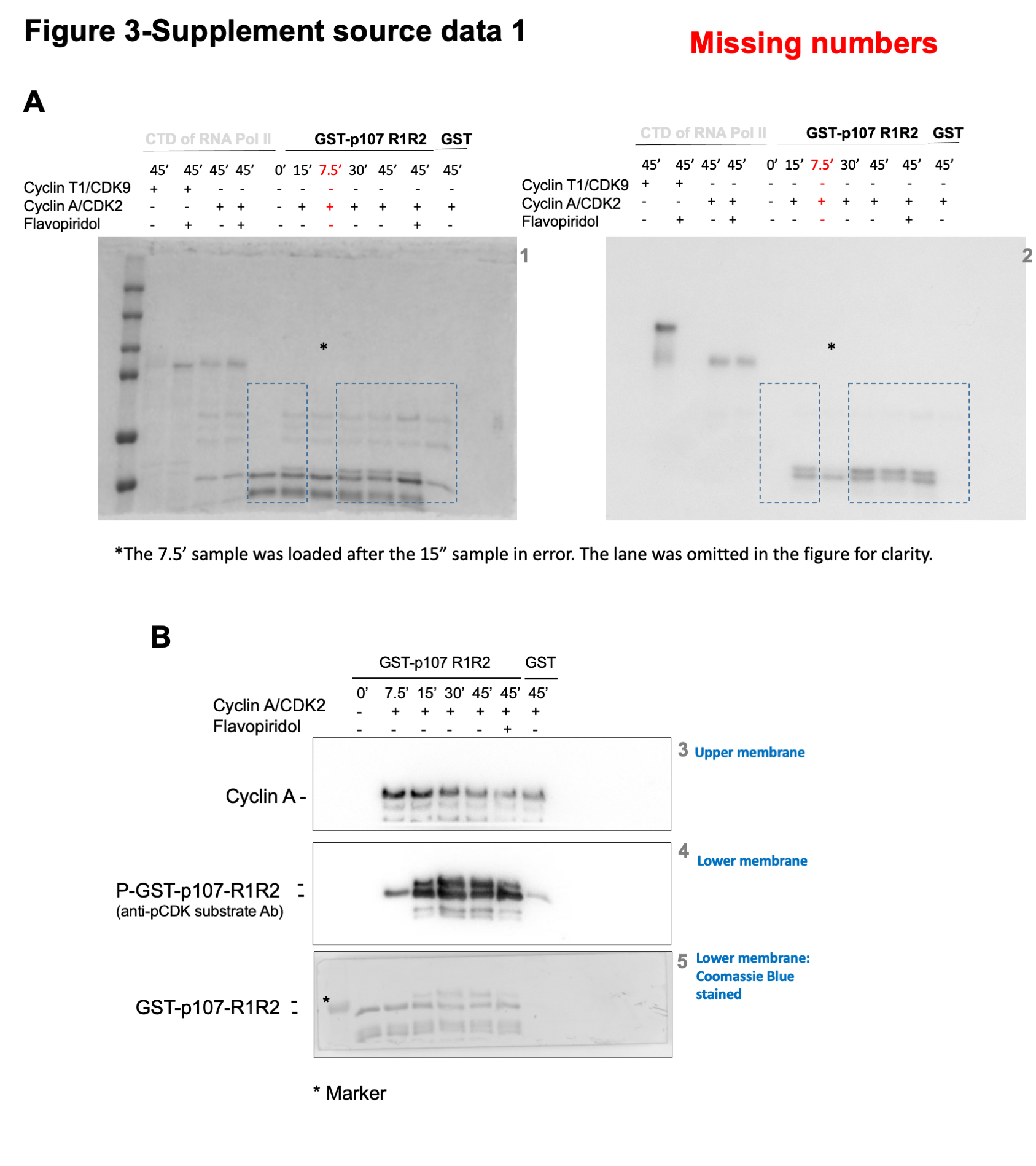

Supplement: Figure 3—figure supplement 1—source data 1. — Dashed boxes correspond to the areas shown in Figure 3—figure supplement 1 (top). The 7.5’ sample was loaded after the 15” sample in error. The lane was omitted in the figure for clarity and the omitted lane marked with an asterisk. [file elife-63181-fig3-figsupp1-data1.zip › e6acef4a-0297-4c59-b820-8ac4abb52563.tiff]

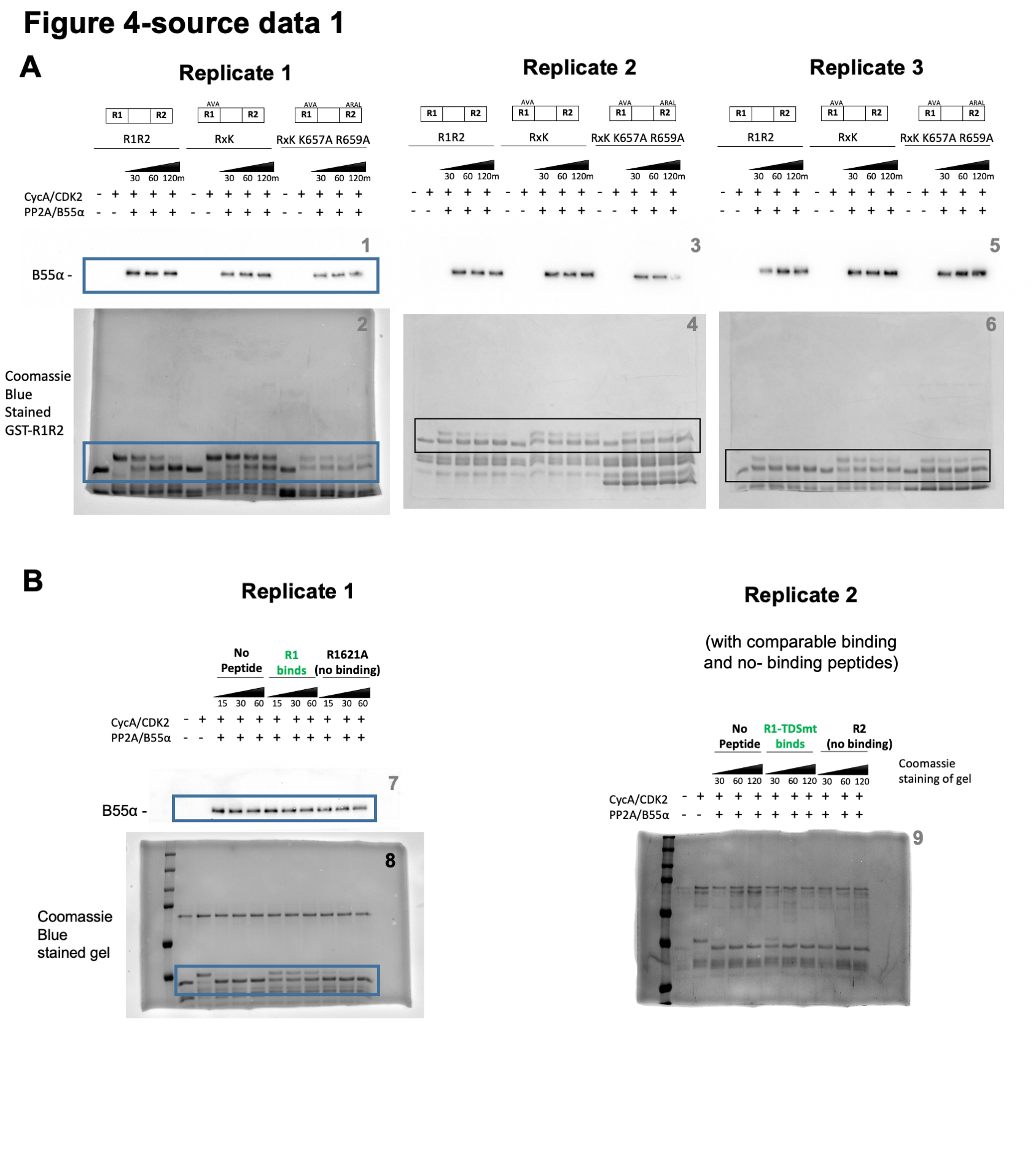

Supplement: Figure 4—source data 1. — Images in Figure 4A were generated from the boxed regions in replicate 1 PVDF membrane and the Coomassie Blue-stained gel. Quantifications shown in Figure 4A of the ‘phospho’-p107 band were obtained from Coomassie Blue-stained gel replicates 1–3. Images in Figure 4B were generated from the boxed regions in replicate 1. A comparable experiment using an R1 peptide (R1-627TDS-AAA) variant that binds B55α (Figure 5B) showed delayed phosphorylation as R1, while R1-R621A and R2, which do not bind B55α (Figure 5A and C), did not inhibit dephosphorylation. [file elife-63181-fig4-data1.zip › 5dda060f-df9f-4e47-a5a2-df280e7c4e20.tiff]

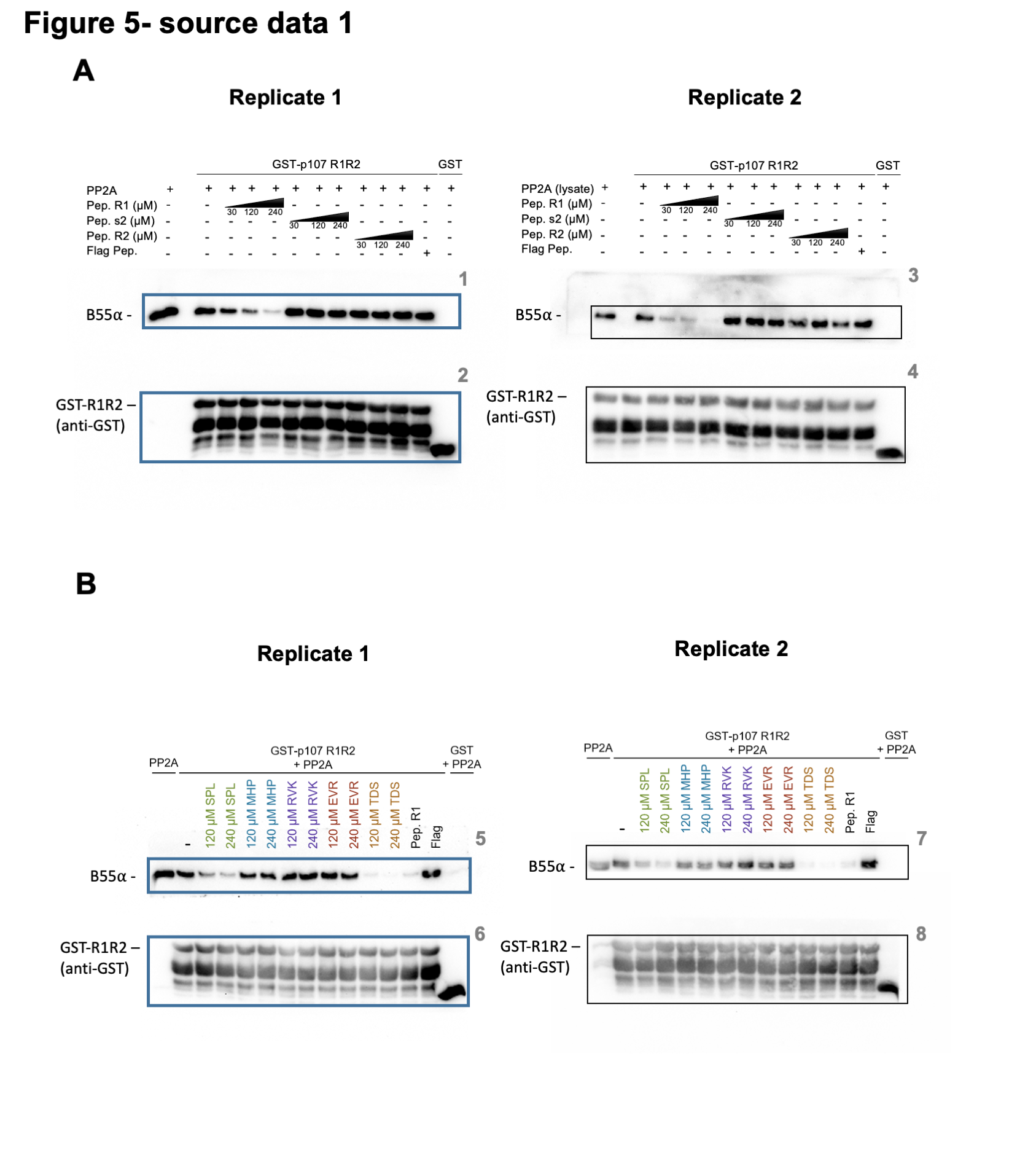

Supplement: Figure 5—source data 1. — Images in Figure 4A and B were generated from the boxed regions in replicate 1 PVDF membranes. B55α band intensities were normalized to the corresponding full-length GST-R1R2 band intensities. [file elife-63181-fig5-data1.zip › 877b4395-01c7-42bf-8127-c2c24826c2fb.tiff]

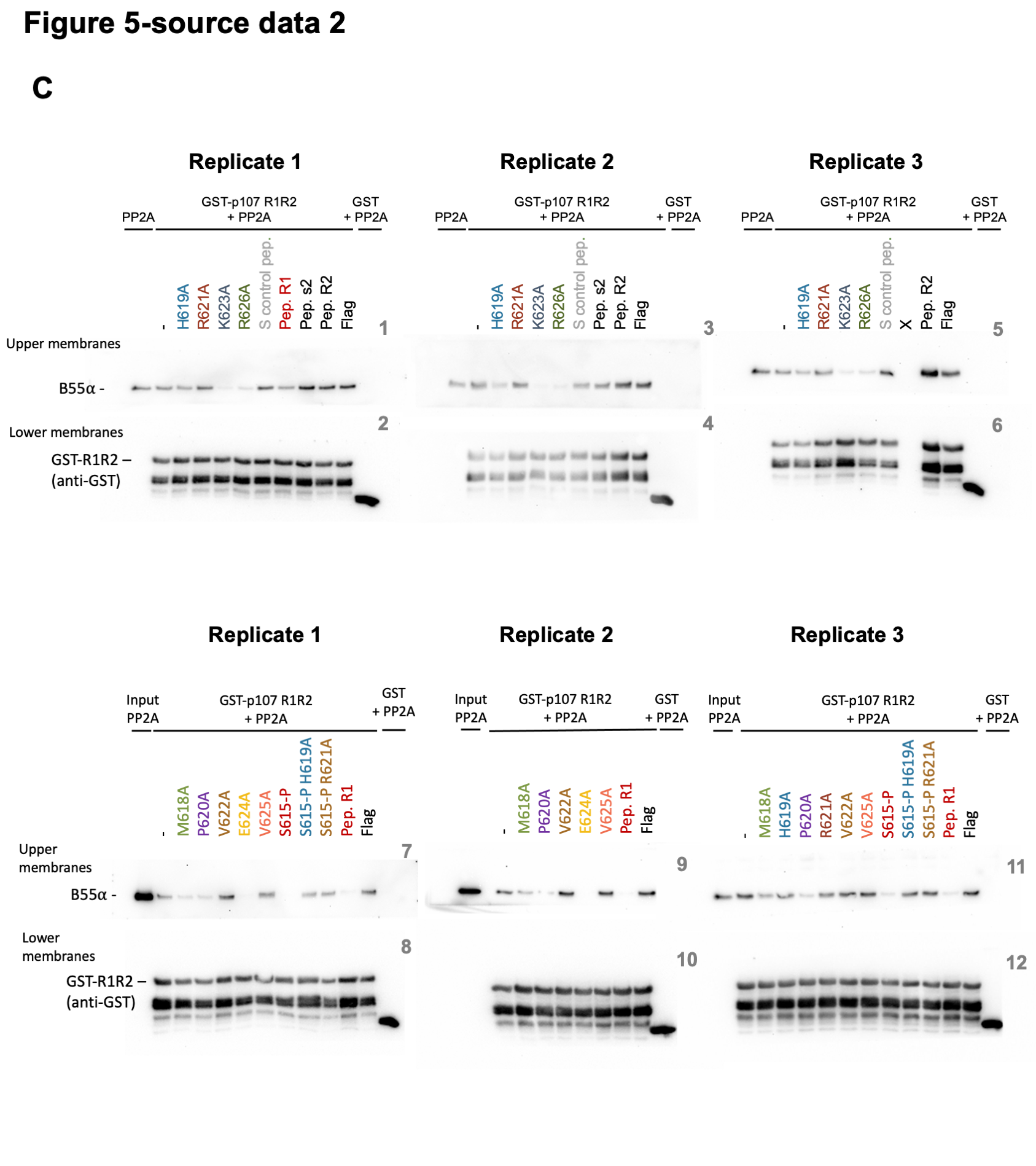

Supplement: Figure 5—source data 2. — B55α band intensities were normalized to the corresponding full-length GST-R1R2 band intensities. [file elife-63181-fig5-data2.zip › f5671292-ca58-4741-9ddf-bf903980740e.tiff]

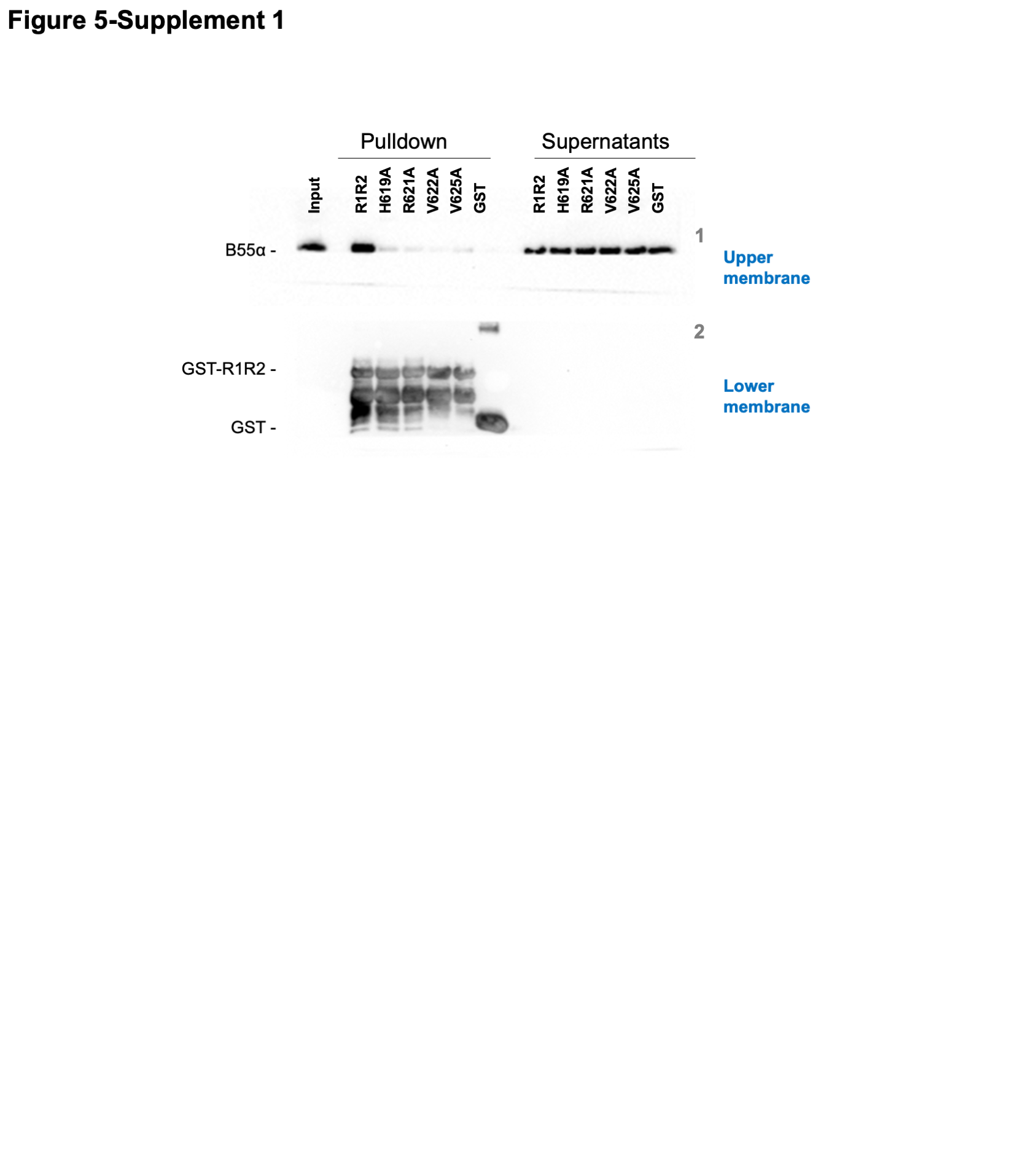

Supplement: Figure 5—figure supplement 1—source data 1. — All the replicates for panel A are shown in Figure 5—source data 2. [file elife-63181-fig5-figsupp1-data1.zip › 7c62eb1e-157c-4c84-b53c-176e1d6d9ed9.tiff]

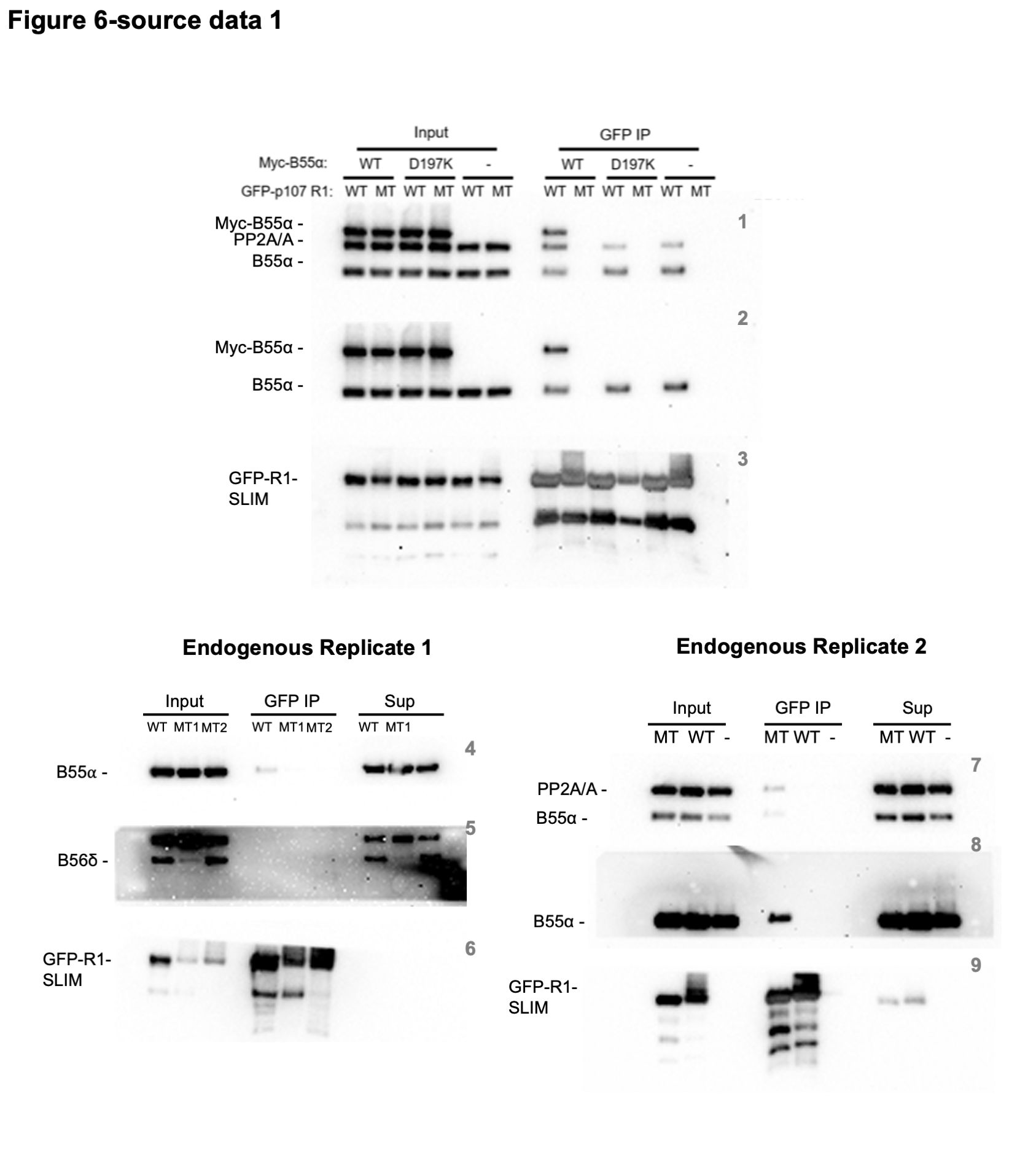

Supplement: Figure 6—source data 1. [file elife-63181-fig6-data1.zip › 13729fb0-5a66-4ca6-b149-40d9e4a6b4a7.tiff]

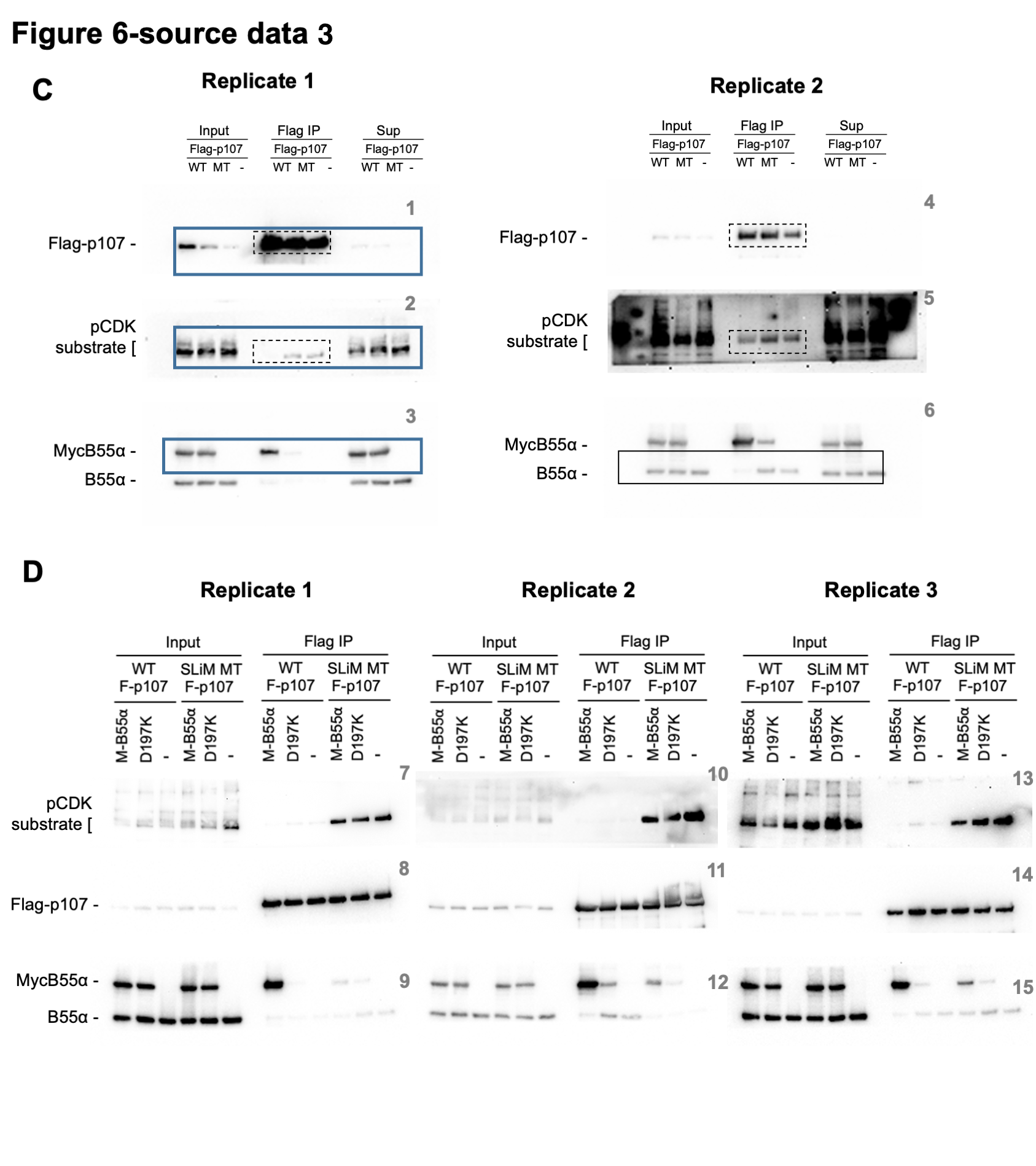

Supplement: Figure 6—source data 3. — In (C), B55α-mediated dephosphorylation was quantitated using the pCDK substrate vs. Flag-p107 signal using the corresponding bands in the dashed boxes. [file elife-63181-fig6-data3.zip › dc58fcde-fee9-4f31-b824-d34798e6b7ca.tiff]
